# New marker for chronic kidney disease progression and mortality in medical-word virtual space

**DOI:** 10.1038/s41598-024-52235-9

**Published:** 2024-01-18

**Authors:** Eiichiro Kanda, Bogdan I. Epureanu, Taiji Adachi, Tamaki Sasaki, Naoki Kashihara

**Affiliations:** 1 Medical Science, Kawasaki Medical School, Kurashiki, Okayama Japan; 2 College of Engineering, University of Michigan, Ann Arbor, MI USA; 3 Institute for Life and Medical Sciences, Kyoto University, Sakyo, Kyoto, Japan; 4 Department of Nephrology and Hypertension, Kawasaki Medical School, Kurashiki, Okayama Japan; 5 Kawasaki Geriatric Medical Center, Okayama, Japan

**Keywords:** Chronic kidney disease, Predictive markers, Applied mathematics, Computer science

## Abstract

A new marker reflecting the pathophysiology of chronic kidney disease (CKD) has been desired for its therapy. In this study, we developed a virtual space where data in medical words and those of actual CKD patients were unified by natural language processing and category theory. A virtual space of medical words was constructed from the CKD-related literature (n = 165,271) using *Word2Vec*, in which 106,612 words composed a network. The network satisfied vector calculations, and retained the meanings of medical words. The data of CKD patients of a cohort study for 3 years (n = 26,433) were transformed into the network as medical-word vectors. We let the relationship between vectors of patient data and the outcome (dialysis or death) be a marker (inner product). Then, the inner product accurately predicted the outcomes: C-statistics of 0.911 (95% CI 0.897, 0.924). Cox proportional hazards models showed that the risk of the outcomes in the high-inner-product group was 21.92 (95% CI 14.77, 32.51) times higher than that in the low-inner-product group. This study showed that CKD patients can be treated as a network of medical words that reflect the pathophysiological condition of CKD and the risks of CKD progression and mortality.

## Introduction

Chronic kidney disease (CKD) is one of the risk factors for cardiovascular disease (CVD), end-stage renal disease (ESRD), and death^1,2^. With the progression of CKD, the risks of these outcomes increase, and complications and comorbidities occur more commonly and become more severe, such as CKD-mineral and bone disorder (MBD), renal anemia, and hyperpotassemia^3^. CKD imposes heavy physical and economic burdens on patients^4,5^. To slow CKD progression, the control of various risk factors is required^6^. The an accurate risk prediction of CKD progression is useful for identifying patients at high risk and evaluating their condition, and it provides us with candidate therapeutic strategies tailored to individual patients^3,7^.

It was pointed out that the number of clinical trials in the field of nephrology has been much lower than that in other medical fields^8^. The main reasons are the complicated pathophysiology of CKD, the difficulty of the trial study design, and the lack of surrogate markers of CKD progression. Recently, the U.S. Food and Drug Administration has proposed the estimated glomerular filtration rate (eGFR) change and eGFR slope as surrogate markers of ESRD^9,10^. However, these surrogate markers are not sufficiently adaptable to various patient conditions and outcomes. Moreover, surrogate markers pose some problems when sampling bias exists, and using the markers created from data from other countries often shows low accuracy. To complement existing markers, there is a need to develop new surrogate markers for various outcomes reflecting the complicated pathophysiology of CKD and patient conditions.

It is very difficult to develop a surrogate marker including the pathophysiology of CKD, because many factors are associated with each other and compose a complex network. Here, it can be considered that medical information is inherent in the medical literature. Thus, the medical literature is a candidate source of data on the pathophysiology of CKD. Moreover, natural language processing (NLP) has been found to be an effective technology for text mining to extract medical concepts, and it has been used in medical research studies^11^. Several studies have shown the relationship of the appearance of medical words such as symptoms and medicines in electronic medical records with the risk of acute kidney injury or ESKD^12^. Recently, various types of artificial intelligence (AI) of NLP have been developed, such as generative pretrained transformer 4 (*GPT-4*), which is used in *ChatGPT*^13^.

*Word2Vec* is an early type of NLP AI and is composed of neural networks for learning word associations from a large corpus of text^14^. This is an efficient tool for obtaining high-quality distributed representations that embed words into vectors^15^. This vectorization enables the calculation of word vectors and the acquisition of meaningful results^15^. It has been reported that semantic information represents objects^16,17^. These lines of evidence suggest that medical-word vectors are expected to compose a network and are associated with real data of CKD patients and the pathophysiological concepts of CKD.

There is difficulty in comparing patient data with data in the medical literature. Category theory is an area of modern mathematics and deals with the relationships between things. Moreover, category theory can link different relationships between medical words and causal relationships between risk factors and outcomes in CKD patients. In cognitive science, research on category theory models has been conducted because the relationship between things can be expressed using functors and the comparison of relationships can be expressed using natural transformations^18,19^. If a medical-word network represents CKD patients’ conditions and prognoses, it could be widely applied to other diseases, e.g., heart disease and cancer.

In previous studies, a medical word was treated as an independent word^12^, on the basis of which we hypothesized that the relationship between medical words related to CKD represents the pathophysiological condition of CKD patients and their prognoses. In this study, we aimed to establish a virtual space of medical words and clarify the following issues on the basis of category theory: (1) CKD-related words compose a network that retains medical concepts and associates with real-world CKD patient data: (2) There is a new surrogate markers for CKD patients’ renal and life prognoses in the virtual space. For this study, data in the medical literature and those of CKD patients were necessary. Data in the CKD-related literature were extracted from MEDLINE, and actual data of CKD patients were obtained from a CKD cohort study in Japan. In addition, to utilize the results of analysis by NLP AI in clinical practice, it is necessary to actually check how NLP AI analyzes medical terminology. Therefore, we purposely used *Word2Vec* rather than the latest NLP AI because its many analysis techniques are publicly available.

## Results

### Distribution of medical-word vectors and calculation

To evaluate the relationships among words, the distributions of the vectors of medical words, *w*s, were examined. The words were found to be distributed near other words with close meanings (Supplementary Fig. 1a). For example, the primary outcome was defined as dialysis or death. *w*_outcome_ located near *w*_esrd_ and *w*_death_,; *w*_ckd_ and *w*_stage_; and *w*_anemia_ and *w*_esa_ (Supplementary Fig. 1b).

The relationships between word vectors could be evaluated on the basis of pairwise cos*θ*. CKD-related words were extracted on the basis of cos*θ* between the vectors (Table 1). *w*_ckd_ was found to be related to CKD progression conditions, such as *w*_esrd_ and *w*_eskd_; the comorbid conditions *w*_cvd_, *w*_dkd_, and *w*_lvh_; and synonyms.

**Table 1:** Medical words related to target words.

Rank	*ckd*		*esrd*		*death*		*ckd* + *diabetes*		*ckd* − *chronic* + *acute*	
	Words	cos*θ*	Words	cos*θ*	Words	cos*θ*	Words	cos*θ*	Words	cos*θ*
1	*esrd*	0.6329	*eskd*	0.7424	*Mortality*	0.6820	*dm*	0.7387	*aki*	0.6146
2	*cri*	0.6241	*esrf*	0.7128	*Dying*	0.6441	*niddm*	0.6670	*arf*	0.4773
3	*cvd*	0.6089	*ckd*	0.6329	*Readmission*	0.5612	*Diabetic*	0.6497	*wrf*	0.4767
4	*crf*	0.6043	*Dialysis*	0.6249	*Rehospitalization*	0.5448	*cvd*	0.6429	*cin*	0.4624
5	*dkd*	0.5839	*cvd*	0.6219	*Demise*	0.5259	*dkd*	0.6265	*ciaki*	0.4438
6	*eskd*	0.5715	*hd*	0.5932	*Died*	0.5193	*iddm*	0.6249	*hf*	0.4422
7	*lvh*	0.5693	*rrt*	0.5861	*Fatality*	0.5088	*esrd*	0.6211	*Hyperkalaemia*	0.4192
8	*chf*	0.5618	*Hemodialysis*	0.5781	*Hospitalization*	0.4898	*Microalbuminuria*	0.6110	*Hyponatraemia*	0.4124
9	*predialysis*	0.5617	*capd*	0.5674	*Mace*	0.4869	*Dyslipidemia*	0.5872	*Hyponatremia*	0.4096
10	*hf*	0.5566	*Haemodialysis*	0.5580	*cve*	0.4841	*Albuminuria*	0.5714	*chf*	0.4079

*cosθ* cosine similarity, *ckd* chronic kidney disease, *esrd* end-stage renal disease, *cri* chronic renal insufficiency, *cvd* cardiovascular disease, *crf* chronic renal failure, *dkd* diabetic kidney disease, *eskd* end-stage kidney disease, *lvh* left ventricular hypertrophy, *chf* chronic heart failure, *hf* heart failure, *esrf* end-stage renal failure, *hd* hemodialysis, *rrt* renal replacement therapy, *capd* continuous ambulatory peritoneal dialysis, mace major adverse cardiac events, *cve* cardiovascular events, *dm* diabetes mellitus, *niddm* non-insulin-dependent diabetes mellitus, *iddm* insulin-dependent diabetes mellitus, *aki* acute kidney injury, *arf* acute renal failure, *wrf* worsening renal function, *cin* contrast-induced nephropathy, *ciaki* contrast-induced acute kidney injury.

This table shows the lists of CKD-related words found on the basis of Cos*θ* of the vectors. The words in italic letters are vectors, e.g., *ckd.*

We then determined the words associated with the results of *w*_ckd_ calculation (Table 1, Supplementary Table 1). The words related to *w*_ckd_ + *w*_diabetes_ were diabetes mellitus (DM) and comorbid conditions. Moreover, the results showed comparatively new concepts: *w*_ckd_ + *w*_diabetes_ = *w*_dkd_; and *w*_ckd_ + *w*_malnutrition_ = *w*_pew_. *w*_aki_ was listed as the first result of *w*_ckd_ − *w*_chronic_ + *w*_acute_.

### Comparison between prognoses made from virtual space and that from real-word data

**Figure 1:**
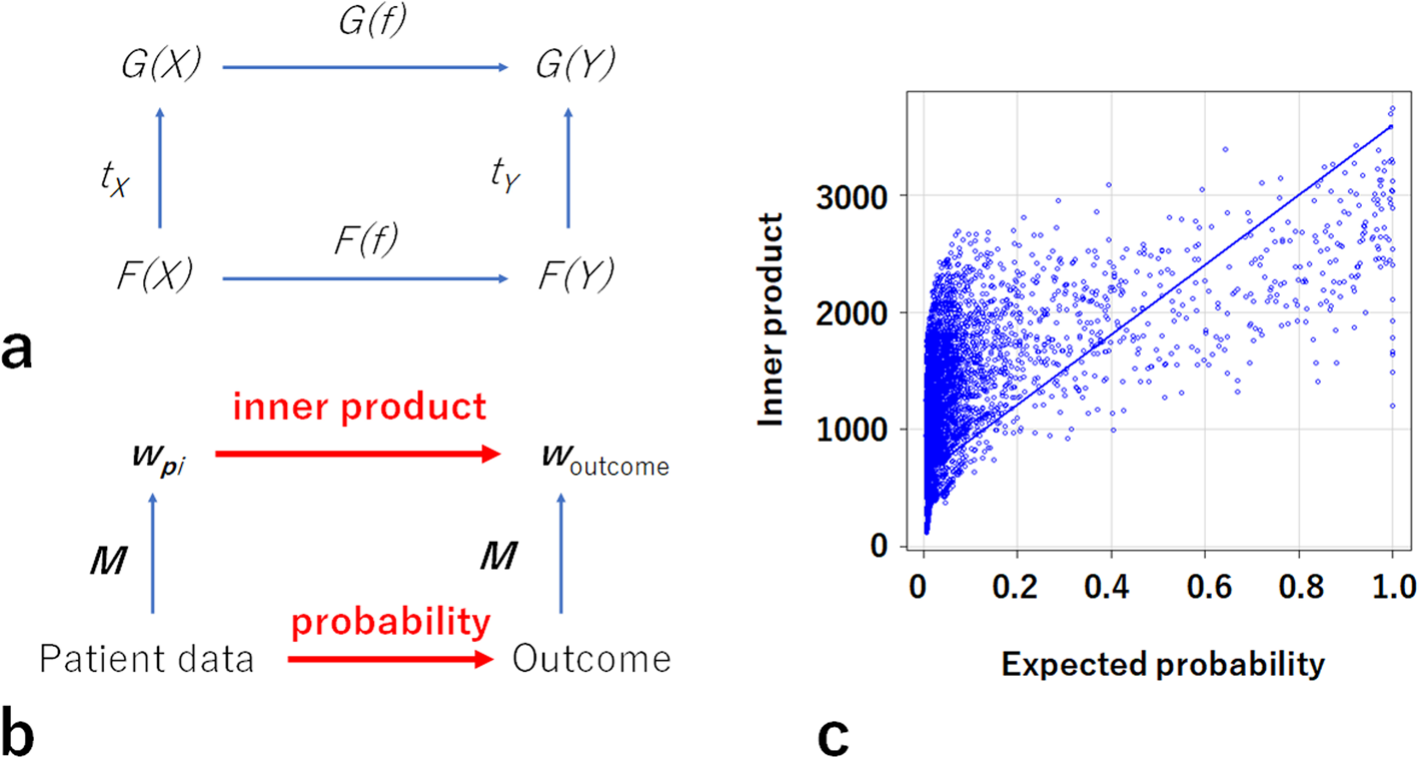
Inner products and predicted probabilities of outcomes. High inner products determined using Model 3 were associated with high probabilities of the outcomes predicted using a logistic regression model. (**a**) Natural transformation *t*: *F* → *G*. (**b**) Analysis of natural transformation in this study. Patient data, outcome, *w*_*pi*_, and *w*_outcome_ correspond to *F(X)*, *F(Y)*, *G(X)*, and *G(Y)*, respectively. The inner products, *G(f)*, were compared with the expected probabilities corresponding to *F(f)*. (**c**) Scatterplot of associations between inner products and expected probabilities.

Table 2 and Supplementary Table 2 show the patients’ baseline characteristics. An inner product and *M* correspond to *G(f)* and *t*, respectively (Fig. 1a,b). It was expected that high inner products are closely correlated with the risks of primary outcomes. The probability of a primary outcome was expected using logistic regression models including the same variables as those in the Models (Supplementary Tables 3, 4). The inner products were found to be statistically significantly associated with the expected probability of the primary outcome using a logistic regression model (LSM) for Model 1, *ρ* = 0.48, *p* < 0.0001; LSM for Model 2, *ρ* = 0.52, *p* < 0.0001; and LSM for Model 3, *ρ* = 0.58, *p* < 0.0001 (Fig. 1c).

**Table 2:** Baseline characteristics.

N	26,433
Demographic characteristic	
Age (years)	61.2 ± 16.4
Male (%)	13,535 (51.2)
Comorbidity	
DM (%)	5537 (20.9)
Hypertension (%)	5442 (20.6)
CVD (%)	171 (0.6)
Laboratory data	
eGFR (mL/min/1.73 m^2^)	73.3 ± 30.8
Albumin (g/dL)	4.2 ± 0.5
Potassium (mmol/L)	4.2 ± 0.4
Phosphorus (mg/dL)	3.3 ± 1.0
LDL (mg/dL)	109.8 ± 32.0
Uric acid (mg/dL)	5.2 ± 1.5
WBC (10^3^/μL)	6.3 ± 3.1
Hemoglobin (g/dL)	13.6 ± 1.9
UPCR (g/gCre)	0.17 [0.11, 0.29]
Medication	
RASI (%)	4578 (17.3)
Phosphorus absorbent (%)	215 (0.8)
Statin (%)	4272 (16.2)
Uric-acid-lowering medicines (%)	2381 (9.0)
ESA (%)	694 (2.6)
Outcome	
Primary outcome (%)	704 (2.7)
ESRD (%)	629 (2.3)
Death (%)	75 (0.3)
Follow-up period (days)	885 [512, 1051]

Continuous variables are shown as mean ± SD or median (interquartile range). Categorical variables are shown as n (%).

*DM* diabetes mellitus, *CVD* cardiovascular disease, *eGFR* estimated glomerular filtration rate, *LDL* low-density lipoprotein, *WBC* white blood cell count, *UPCR* urinary protein-to-creatinine ratio, *RASI* renin–angiotensin–aldosterone system inhibitor, *ESA* erythropoietin-stimulating agent, *ESRD* end-stage renal disease.

### Relationships between inner products and outcomes

**Figure 2:**
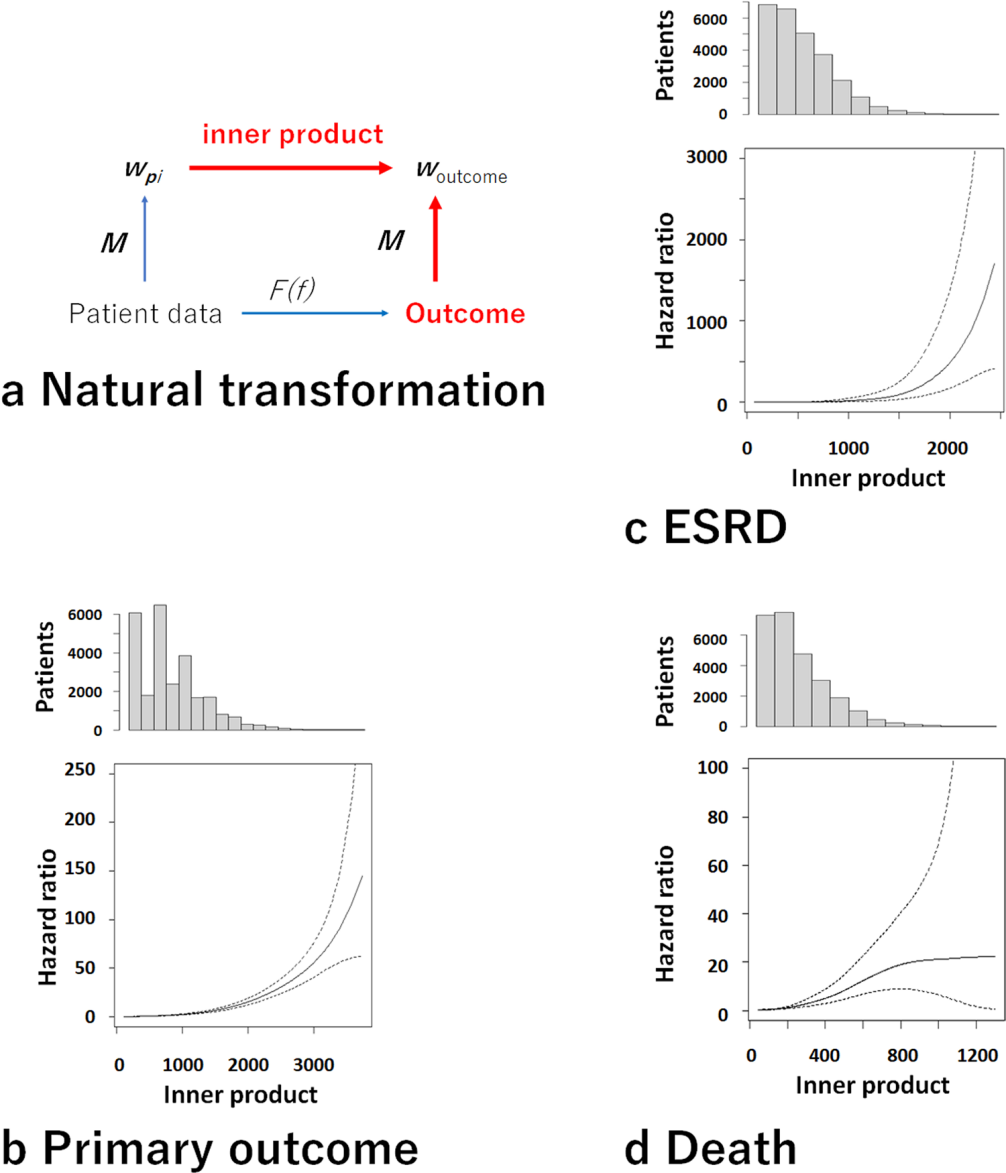
Relationships between inner products obtained using Model 3 and outcome risks. (**a**) Analysis of natural transformation. It was evaluated whether the inner products were associated with the risks of the outcomes. (**b–d**) Relationships between the inner products evaluated on the basis of Model 3 and the risks of the outcomes were observed (*p* < 0.0001, respectively). Histograms of the inner products and the hazard ratios of the outcomes are shown in the upper and lower panels, respectively.

The relationships between *G(f)* and the outcomes were evaluated using Cox proportional hazards models (Fig. 2a). The patients with high inner products showed high risks of the primary outcomes (*p* < 0.0001) (Fig. 2b). The high inner products were also associated with high risks of ESRD and death (Fig. 2c,d). Models 1 and 2 showed similar trends (Supplementary Fig. 2).

Moreover, the relationships between the inner products and the primary outcomes were also evaluated on the basis of the receiver operating characteristic (ROC) curve (Supplementary Fig. 3). The C-statistics for the prediction of the outcome using Model 3 was 0.911 (95% CI 0.897, 0.924).

### Subclass analysis

The relationships between the inner products and the outcomes were evaluated using Model 3 in subclasses on the basis of DM and age (Supplementary Fig. 4). Cox proportional hazards models showed that high inner products were associated with high risks of the outcomes in the DM and non-DM groups (*p* < 0.0001) (Supplementary Fig. 4a,b). The young and old groups showed similar trends (*p* < 0.0001) (Supplementary Fig. 4c,d). Moreover, similar relationships were also observed in the high-eGFR, low-eGFR, proteinuria-negative, and proteinuria-positive groups (*p* < 0.0001) (Supplementary Fig. 5).

### High-risk group identified on the basis of inner products

**Figure 3:**
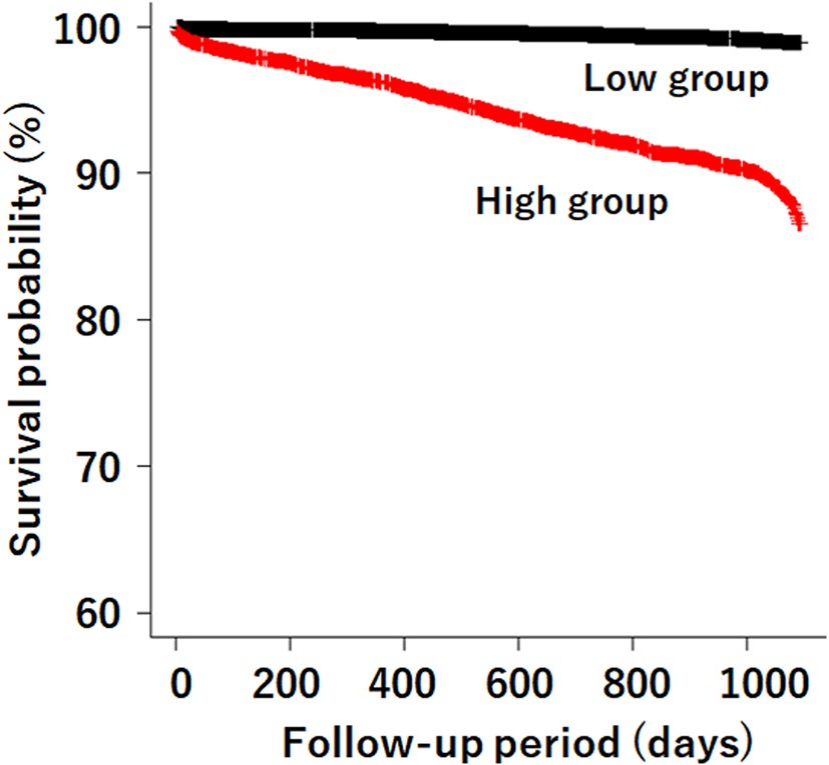
Survival probabilities of inner-product groups. Kaplan–Meier curves show that the survival probability of the high-inner-product group was lower than that of the low-inner-product group (Log-rank test, *p* < 0.0001). *High group* high-inner-product group, *Low group* low-inner-product group.

For the inner products of Model 3 to be informative and show whether a patient was at high risk or not, the cutoff level of the inner products for the prediction of an outcome was determined to be 965 on the basis of the ROC curve (Supplementary Fig. 3). Kaplan–Meier survival curves revealed that the high-inner-product group showed lower survival probabilities than the low-inner-product group (Fig. 3). The hazard ratio (HR) of the outcome of the high-inner-product group was 21.92 (95% CI 14.77, 32.51). Likewise, the HRs of ESRD and death were 4.09 (95% CI 2.58, 6.50) and 13.85 (11.20, 17.12), respectively.

### Entire structure of virtual space

**Figure 4:**
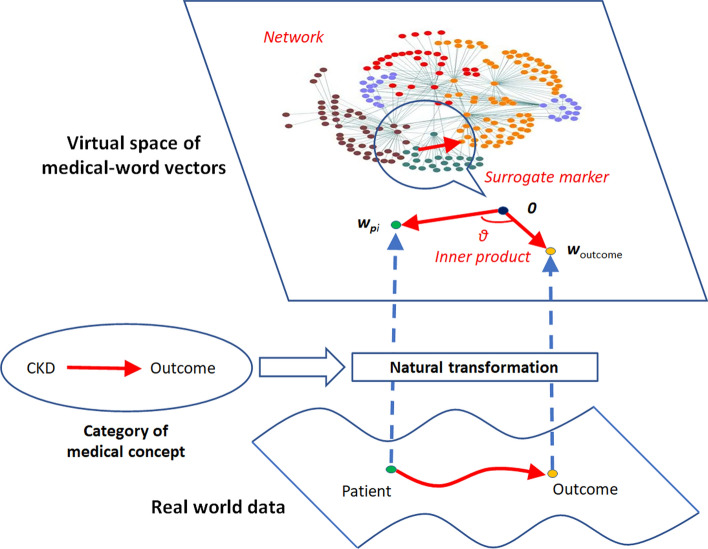
Illustration of virtual space. The real-world data of CKD patients are mapped to the virtual space of medical-word vectors on the basis of natural transformation that retains medical concepts. The virtual space forms a network of medical concepts, where the origin 0 and the outcome vector *w*_outcome_ are defined as standards. *w*_*pi*_, patient vector.

Figure 4 shows an illustration of the relationships between the real-world data of CKD patients and the virtual space of medical-word vectors. The natural transformation, which retained the semantic relationships between the CKD-related factors, mapped patient data into the virtual space where the inner products were in agreement with patients’ prognoses. Therefore, these inner products were useful as a surrogate marker for the risk evaluation of CKD patients.

## Discussion

In this study, we created vectors of medical words from the medical literature and focused on the relationships between these vectors. Moreover, the relationships are kept in patients. In other words, it was shown that a patient can be treated as a kind of sentence of medical terms and that this text information is related to the prognoses of life and kidneys. In this process, we found that the inner product becomes a surrogate marker for renal and life prognoses. Therefore, these results taken together suggest that the medical-word network universally represents the network of various pathophysiological factors for diseases.

This study showed that the inner product led to the accurate prediction of the renal and life prognoses of CKD patients, suggesting that it can be a new surrogate marker for CKD patients’ prognosis. The marker is useful in clinical settings in, e.g., risk estimation, diagnosis, clinical therapy decision, and specialist referral^20^. Moreover, our study showed that it is possible to convert the inner product into a substitute indicator of event occurrence probability on the basis of the relationship between the inner product and the probability of event occurrence. In previous clinical studies, changes in patient characteristics and data were observed owing to the use of certain drugs. Therefore, by calculating the inner product before and after treatment, it is possible to calculate the change in the inner product, which can be used to evaluate the change in the probability of event occurrence. Furthermore, if it becomes possible to add new drugs as variables, it may be possible to apply a more accurate AI technology to clinical studies.

Various models for risk prediction for CKD patients have been developed for a target population. Thus, the models cannot always show high prognostic accuracies when they are applied to other populations^7,20,21^. One of the reasons for the inaccuracies is selection bias. Here, the inner product is defined as the length of a patient vector mapped on the outcome vector: ${||w}_{p}||{\text{cos}}\theta \cdot |\left|{w}_{{\text{outcome}}}\right||$. Thus, when $|\left|{w}_{{\text{outcome}}}\right||$ was considered a unit for measuring a patient’s condition, such as meters and kilograms, ${||w}_{p}||{\text{cos}}\theta$ reflected the patient’s condition status. In the virtual space, the conditions of patients in any population were evaluated using the same measure $|\left|{w}_{{\text{outcome}}}\right||$; thereby, the errors of the risk prediction due to the difference in populations were minimized. Thus, the inner product is useful and robust for risk prediction.

To confirm the relationship between vectors, nephrologists actually and visually confirmed the distribution and relationship of words. It was also confirmed that vector operations can be performed while maintaining medical meanings, so it is possible to treat patients as vector-like entities that hold the relationship between medical terms. Next, we showed that the causal relationship between the patient’s condition and prognosis holds in both the medical term virtual space and the real world. Since category theory is a field that treats relationships between things similarly to sets, the universality of the causal relationship between the patient’s condition and prognosis was demonstrated by evaluating the relationship based on natural transformations of category theory. From the above, it was found that treating patients similarly to sentences enables the prediction of their prognosis. Moreover, new applications of category theory to medical fields such as internal medicine and cardiology have been demonstrated by utilizing category theory in nephrology as well as cognitive science.

It has been reported that when words such as symptoms appear in electronic medical records, the risk of acute kidney injury or end-stage kidney disease increases^12^. Our study is consistent with these research results. Furthermore, by focusing on the relationship between medical terms and vector operations on patient data, we believe that data science technology has become easily applicable to the evaluation of patients’ conditions and prognoses. Thus, even without patients’ data such as blood test results, it may be possible to easily determine the degree of risk by entering information such as age, gender, diabetes, and hypertension into chatbots using cutting-edge NLP AI models such as *GPT-4*.

Although the inner product calculation in this study is very complex, it can be easily put into practical use using information and communication technology. We previously developed an AI algorithm that predicts the prognosis of CKD patients and made it available for actual use^22^. In this system, when a user inputs patient data, the AI algorithm on the server automatically calculates and displays the risk. A system that incorporates this AI algorithm into the server will enable users to use this system on their internet browser without having to perform complex inner product calculations.

Currently, NLP AI models such as *GPT-4* are rapidly developing and can be used on the internet on a daily basis. To utilize such technologies in clinical medicine, it is necessary to confirm the type of analysis being performed. In this study, we used *Word2Vec* to extract word vectors, which is the progenitor leading to the latest NLP AI models and can be treated more easily than the latest NLP AI models^14^. It was confirmed clinically that the analysis of medical literature by NLP AI is reliable, as nephrologists actually verified the analysis results. Verification using models other than *Word2Vec* is a future research task.

The other contribution of our study is that the medical words formed a network on the basis of similarities between them. Moreover, the network and actual interobject relationships can be linked to each other on the basis of the natural transformation of category theory. A word network can be used to assist pathologists or physicians in evaluating the genetic relatedness of diseases^23^. As an example, in our preliminary study, we newly found the relationship between CKD and zinc using the prototype of the network and confirmed this finding using CKD cohort data^24^. These lines of evidence indicate a strong potential of the network for medical research. Thus, the network can provide a new disease model on the basis of unified medical knowledge as a new tool. For example, a list of various factors for the target disease includes medicines, risk factors, genes, and molecules. It can lead to finding new research seeds, which usually takes a long time and much effort to realize and require the reading of thousands of papers. Moreover, the network is applicable to the pathophysiological conditions of not only CKD but also other diseases such as CVD and cancer.

Our models are applicable to clinical settings in the context of their limitations as follows. First, only some of the medical words clinically related to CKD were evaluated. Although it is impossible to evaluate the relationships among all medical words, it is necessary to confirm the words related to other diseases and molecular biology. Second, the CKD patient data in Japan were used. Future studies using data from patients from other countries and other diseases are required for external validations. It will be medically beneficial to show the network of medical words related to other diseases, such as non-CKD, heart failure, and cancer. Third, we analyzed text data obtained from PubMed and confirmed the medical meanings of the obtained words with three nephrologists. At this time, inappropriate words with wrong spellings or forms were removed. This confirmation work may have low reproducibility. However, since the words were checked by the nephrologists, there was no problem in terms of medical definition. Fourth, albuminuria is an important risk factor related to the progression and prognosis of CKD. However, in Japan's health insurance system, albuminuria can only be tested for diabetic patients. Therefore, in this study, we were unable to investigate albuminuria as a variable and used proteinuria instead. Furthermore, to make the model generally available, we considered the urinary protein-to-creatinine ratio (UPCR) as a continuous variable, not a categorical variable. It has been reported that urine albumin-to-creatinine ratio (UACR) is estimated from UPCR using Weaver et al.’s estimation formulae^25^. Since UACR and UPCR can be numerically converted, our model is considered valid even if UACR is used. Variables related to CKD, including UACR, are subjects for future studies. Fifth, in our model, urinary protein was input as a continuous variable. Normalization, logarithmic transformation, and categorical variable conversion are some of the transformations used for urinary protein, which show a skewed distribution. However, these transformations include some issues such as sampling bias and problems with calculation methods and the determination of medical cutoff values. Therefore, in this study, we used UPCR as it circumventss these issues. The optimal transformation of variables related to CKD, such as phosphorus and parathyroid hormone levels, is a topic for future research.

## Conclusions

This study showed the associations between the virtual space of medical words and CKD patients’ data, on the basis of which we proposed a new surrogate marker of renal and life prognoses of CKD patients. Our results also suggest that the virtual space reflects the pathophysiological condition of patients and that NLP AI technologies can predict patient prognosis.

## Methods

### Virtual space constructed on the basis of category theory and analysis steps

CKD is defined as the abnormalities of the kidney structure or function with complications, such as renal anemia, hyperkalemia, and mineral and bone disorders. CKD progression leads to ESRD and death. Here, we use the meanings of words as a functional structured database of actual objects. The meanings of words could be treated as a mathematical set^26,27^.

A category is a collection of objects with morphisms (arrows) between them^28,29^. The concept of relationships between the deterioration of kidney function with comorbid conditions and outcomes, such as ESRD and death, can be considered as a morphism from CKD to outcomes (*X* → *Y*), and these factors compose category $\mathcal{A}$ (Supplementary Fig. 6). These relationships are also observed in real data of CKD patients and descriptions in papers about CKD. When we let a natural transformation in $\mathcal{B}$ be *t*: *F* → *G*, the relationships between CKD and outcomes in the patient data were connected with those in the papers. These relationships composed a virtual space that included data from CKD patients and data from the medical literature as vectors.

These relationships were also observed in real data of CKD patients and descriptions in papers about CKD, which were treated as categories ${\mathcal{B}}_{1}$ and ${\mathcal{B}}_{2}$ in this study, respectively. Morphisms between categories are called functors. In these relationships, functors *F* and *G* connected $\mathcal{A}$ with ${\mathcal{B}}_{1}$ and ${\mathcal{B}}_{2}$, respectively (Supplementary Fig. 6a). A natural transformation involves a family of morphisms. When ${\mathcal{B}}_{1}$ and ${\mathcal{B}}_{2}$ were composed of vectors, these categories were summarized into category $\mathcal{B}$ (Supplementary Fig. 6b). When we let a natural transformation in $\mathcal{B}$ be *t*: *F* → *G*, it linked the relationships between CKD and outcomes in the patient data and those in the medical words. $\mathcal{B}$ could be considered as the virtual space of medical-word vectors.

### Analysis steps

The major analysis steps shown in Supplementary Fig. 7 are as follows: (1) download abstracts of medical papers from MEDLINE using PubMed; (2) create the virtual space *W* of medical-word vectors and the matrix *M* for the linear transformation of patient data using NLP; (3) evaluate the medical and mathematical characteristics of *W*; (4) map the CKD patient data into the virtual space as patient vectors using *M*; and (5) compare the patient vectors with actual prognoses.

### NLP of medical text data

Medical papers about CKD were searched using PubMed with the query “(CKD) OR (chronic kidney disease) OR (CRF) OR (chronic renal failure)”. We also used the following filters: language, English; published until the end of 2019. A total of 165,271 papers were available, from which their titles and abstracts were extracted (Supplementary Fig. 7). Texts were preprocessed by formatting words into lowercase letters, tokenization, lemmatization, stop word removal, and lemmatization using the default settings of *Natural Language Toolkit* (*NLTK*) package 3.8.1 of Python. Medical words have problems with their acronyms and synonyms because the selection of medical words affects their vectors and results. Moreover, in NLP, continuous words are analyzed separately. For example, CKD is an acronym for chronic kidney disease and is synonymous with chronic renal failure (CRF). To overcome these problems, three nephrologists manually confirmed all words using medical dictionaries^30–32^ and selected commonly used single words or acronyms at present, considering that medical words recently used reflect new concepts and that acronyms are more often used in a paper than strings of words. As a result, 106,612 words were retained.

Word embedding enables words and phrases to represent vectors of numeric values with low and high dimensions. We adopted a continuous-bag-of words (*CBOW*) model. Untrained *Word2Vec* 0.11.1 in *Gensim* 4.3.2 of Python was used to create a 200-dimensional (D) word vector ***w*** in the vector space *W* for each medical word^33^. The parameters of *Word2Vec* were as follows: size = 200, alpha = 0.025, min_count = 5, window = 5, workers = 3, and epochs = 5. The word vectors, ***w***s, were saved, loaded, and extracted from the model developed using *Word2Vec*.

### Evaluation of characteristics of medical-word network in virtual space

The vectors were distributed in a 200-D space. Their distribution was plotted in a 2-D space after dimension reduction by t-distributed stochastic neighbor embedding (t-SNE). Cosine similarities (cos*θ*) were useful for finding related words in *W*. The relationships between word vectors, *w*, were evaluated on the basis of pairwise cos*θ*:

1 $$\begin{array}{c}cos\theta = \frac{{w}_{k}{ \cdot w}_{l}}{\Vert {w}_{k}\Vert \Vert {w}_{l}\Vert } for\,k, l\in \left(\mathrm{1,2},\dots \right),\end{array}$$

where cos*θ* = 1 means extremely similar and cos*θ* =  − 1 means the opposite. Generally, vectors can be calculated by, for example, addition, subtraction, and scalar multiplication. Then, to investigate whether the result of vector calculation could retain the expected meanings, the word vectors related to a vector of CKD, *w*_ckd_, were extracted using cos*θ*.

### Study design and population

A three-year cohort study was conducted on CKD patients who visited Kawasaki Medical School Hospital, Japan from January 1st, 2018 to December 31st, 2020 (CKD cohort study). Patients were eligible for inclusion in the cohort when they were at least 20 years of age and diagnosed as having CKD on the basis of the criteria of the Japanese Society of Nephrology^2^. We treated CKD in accordance with the CKD clinical practice guideline of the Japanese Society of Nephrology^2^. The present study included the data from the CKD cohort study (n = 67,957) (Supplementary Fig. 7). The exclusion criteria were as follows: patients on any type of dialysis, patients who had malignancies, or patients who had no eGFR data. Thus, 26,443 patients were included in the analysis and followed up during the study period. This study was approved by the Research Ethics Committee of Kawasaki Medical School (No. 5306-1, 6047-00). The exemption for informed consent from participants is also approved by the Research Ethics Committee of Kawasaki Medical School. The study was performed in accordance with the relevant guidelines and the Declaration of Helsinki.

### Variables

Variables in this study were selected on the basis of common measurements at outpatient clinics, namely, CKD-related factors and medications^3,34–37^. The data were extracted from an electronic-medical-record-data server in Kawasaki Medical School Hospital. The variables were as follows (Supplementary Table 4): age; gender; DM; hypertension; history of CVD; eGFR; serum albumin, potassium, phosphorus, low-density lipoprotein, and uric acid levels; white blood cell count; hemoglobin level; UPCR; and use of renin–angiotensin–aldosterone system inhibitors (RASIs), phosphorus absorbents, statins, uric-acid-lowering medicines, and erythropoietin-stimulating agents. eGFR was calculated using the equation for the Japanese population^38^. The variables with more than 20% missing values of data were not included in the analysis. After typing of missing values, multiple imputation was conducted appropriately to account for missing data in analyses.

Most of the variables were categorized into binary variables on the basis of the CKD clinical practice guidelines of the Japanese Society of Nephrology^2^. Seventeen variables were used in analyses as follows (Supplementary Table 4): elderly, male, CKD stage, proteinuria, CVD, DM, hypertension, dyslipidemia, hypoalbuminemia, inflammation, anemia, hyperkalemia, hyperphosphatemia, hyperuricemia, and use of RASIs.

The primary outcome was defined as ESRD or any-cause death within 3 years. ESRD was defined as CKD stage G5, the initiation of renal replacement therapy, or kidney transplantation. However, none of the patients in this study had kidney transplantation. When no outcome was observed within the observation period, the observation was treated as a censored one. The incidences of ESRD and death were also evaluated as secondary outcomes. In this study, each word vector is shown in italic letters, for example, *w*_outcome_. The vector of an outcome was defined as *w*_outcome_ = *w*_esrd_ + *w*_death_.

### Statistical analyses

The statistical data are shown as mean ± SD for normal distribution; otherwise, the median and interquartile ranges are presented. All analyses were carried out using SAS version 9.4 (SAS Institute, NC, USA), Python 3.8.2 (Python Software Foundation, DE, USA), and R version 3.6.1 (R Foundation for Statistical Computing, Vienna, Austria). Statistical significance was defined as a two-sided *p* < 0.05.

### Linear transformation of patient data to virtual space

The characteristics of a patient (*i*) were linearly transformed as a vector *w*_*pi*_ to the virtual space *W* using a matrix *M* composed of medical-word vectors *w* (Supplementary Fig. 8).

A patient $i\in (\mathrm{1,2},\dots )$ had the $n\in (\mathrm{1,2},\dots )$ variables of characteristics, such as age and gender. These variables (v_*i*1_, …, v_*in*_) are expressed as a vector, ${v}_{i}\in {\mathbb{R}}^{n}$. ${v}_{i}$ is written as

2 $$\begin{array}{c}{{\varvec{v}}}_{i}={{\text{v}}}_{i1}\left(\begin{array}{c}1\\ \vdots \\ 0\\ \vdots \\ 0\end{array}\right)+\dots +{{\text{v}}}_{ij}\left(\begin{array}{c}0\\ \vdots \\ 1\\ \vdots \\ 0\end{array}\right)+\dots + {{\text{v}}}_{in}\left(\begin{array}{c}0\\ \vdots \\ 0\\ \vdots \\ 1\end{array}\right)\,for\, j\in \left(\mathrm{1,2},\dots \right).\end{array}$$

The set $$\left(\begin{array}{c}1\\ \vdots \\ 0\\ \vdots \\ 0\end{array}\right),\dots ,\left(\begin{array}{c}0\\ \vdots \\ 0\\ \vdots \\ 1\end{array}\right)$$ forms a basis of $${e}_{j}=\left(\begin{array}{c}0\\ \vdots \\ 1\\ \vdots \\ 0\end{array}\right) \in {\mathbb{R}}^{n}$$.

Because each variable of patient characteristics as a medical word has a vector *w*_*j*_, the linear transformation *α*_*j*_: *e*_*j*_ → *w*_*j*_ is conducted using the matrix *M*. The patient vector *w*_*pi*_ is as follows:

3 $$\begin{array}{c}{w}_{pi}={{\text{v}}}_{i1}{w}_{1}+\dots +{{{\text{v}}}_{ij}w}_{j}+\dots +{{\text{v}}}_{in}{w}_{n},\end{array}$$

$${w}_{pi}=M\left(\begin{array}{c}{{\text{v}}}_{i1}\\ \vdots \\ {{\text{v}}}_{in}\end{array}\right),$$

where *M* is the matrix composed of *w*_*j*_. *M* induces the linear transformation ${\mathbb{R}}^{n}\to {\mathbb{R}}^{200}$, and transforms the patient data into *W* (Supplementary Fig. 8).

When a patient had a characteristic, its vector was added to the sum of vectors. *w*_ckd_ was assigned to a patient whose eGFR was less than 60 mL/min/1.73 m^2^. *w*_ckd_ + *w*_stage_ and *w*_ckd_ + 2 × *w*_stage_ were assigned to patients with CKD stages G4 and G5, respectively. The origin vector *0* was defined as a patient who had no risk factors such as CKD stage G1 or G2, proteinuria, or other characteristics. Regarding gender, because male or female cannot be treated as *0*, *w*_male_ or *w*_female_ was assigned to *w*_*pi*_.

To evaluate the effects of risk factors on outcome risks, we constructed three models as follows:

$$\begin{aligned} {\text{Model 1}},w_{pi} & = w_{{{\text{elderly}}}} + w_{{{\text{male}}}}\,{\text{or}}\,w_{{{\text{female}}}} + w_{{{\text{ckd}}}} + w_{{{\text{stage}}}} + {\text{ proteinuria level }}\left( {{\text{g}}/{\text{gCr}}} \right) \, \\ & \quad\times w_{{{\text{proteinuria}}}},\end{aligned}$$

$$\begin{aligned} {\text{Model 2}},w_{pi} & = {\text{ Model 1 }} + w_{{{\text{cvd}}}} + w_{{{\text{diabetes}}}} + w_{{{\text{hypertension}}}} + w_{{{\text{dyslipidemia}}}} \\ &\quad+ w_{{{\text{hypoalbuminemia}}}} + w_{{{\text{inflammation}}}},\end{aligned}$$

$$\begin{aligned} {\text{Model 3}},w_{pi} & = {\text{ Model 2 }} + w_{{{\text{anemia}}}} + w_{{{\text{hyperkalemia}}}} + w_{{{\text{hyperphosphatemia}}}} + w_{{{\text{hyperuricemia}}}} \\ &\quad+ w_{{{\text{rasi}}}}.\end{aligned}$$

### Inner products between patient vector and outcomes

We assumed that both the primary outcomes, ESRD and death, would co-occur with risk factors in papers in MEDLINE and that the representation of risk-factor vectors in the word-embedding space would be similar to that of the outcomes on the basis of the contextual nature of CBOW modeling.

The cos*θ* between *w*_*pi*_ and *w*_outcome_ could be used for evaluating the relationship between them. However, the *θ* between vectors sometimes became larger instead of a risk-factor vector being added (Supplementary Fig. 9a). Thus, inner products between *w*_*pi*_ and *w*_outcome_ were used to evaluate the prognoses of a patient. An inner product referred to the product of the norms of vectors, such as the projection of *w*_*pi*_ on *w*_outcome_ and that of *w*_outcome_ (Supplementary Fig. 9b). Here, the norm of *w*_outcome_ was constant. That is, the norm of *w*_*pi*_ on *w*_outcome_ could be considered as an index of a patient’s condition.

### Inner product and risk of outcome

Considering the definition of natural transformation, the square of commutes (Fig. 1a)

4 $$\begin{array}{c}G\left(f\right)^\circ {t}_{x}= {t}_{y}^\circ F\left(f\right).\end{array}$$

An inner product and *M* correspond to *G(f)* and *t*, respectively (Supplementary Fig. 6b). The patient data were mapped to the virtual space by natural transformation based on category theory, where the patient data could be calculated mathematically.

*Analysis step (1)* A patient’s risk of the outcomes as *F(f)* was evaluated using the probability *p*_*i*_ of the outcome occurrence predicted using a multivariate LRM including the same baseline characteristics as those in Models 1 to 3 (Supplementary Table 4):

5 $$\begin{array}{c}{p}_{i} = \frac{1}{1+{\text{exp}}\left(-{\sum }_{0}^{n}{\beta }_{m}{v}_{m}\right)}for\,m\in \left(0, 1,\dots , n\right).\end{array}$$

LRM for Model 1 included elderly, male, ckd, stage, and proteinuria level (g/gCr). LRM for Model 2 included variables in LSM for Model 1, cvd, diabetes, hypertension, dyslipidemia, hypoalbuminemia, and inflammation. Model 3 included variables in LSM for Model 2, anemia, hyperkalemia, hyperphosphatemia, hyperuricemia, and rasi. The expected probabilities of the outcomes were compared with the inner products using Spearman’s rank correlation coefficients (*ρ*).

*Step (2)* To compare the inner products with the risks of the outcomes, univariate Cox proportional hazards models including the spline of the inner products were used. The results are presented as HR with 95% CI.

A cutoff level of each model was determined on the basis of the Youden index on the ROC curve for the prediction of the outcome. The patients were categorized into high- and low-inner-product groups. The risks of the outcomes were compared between the groups using Kaplan–Meier survival curves and Cox proportional hazards models. Moreover, in the analysis of competing risks of ESRD and death, Fine and Gray competing risk regression models were used. Then, the applicability of the models to a subclass of patients was evaluated on the basis of DM, age (young, younger than 65; old, 65 or older), eGFR (high, 60 mL/min/1.73 m^2^ or higher; low, less than 60 mL/min/1.73 m^2^), and proteinuria (negative, 0.15 g/gCr; positive, 0.15 g/gCr or higher), using data from the cohort study of CKD patients.

### Supplementary Information

Supplementary Information.

## Data Availability

The patient data used in this study will be provided upon reasonable request to Kawasaki Medical School Hospital.
