# Supplementary material for: New marker for chronic kidney disease progression and mortality in medical-word virtual space
Source: Sci Rep. 2024 Jan 18;14:1661. doi: 10.1038/s41598-024-52235-9 (PMC10796328; doi:10.1038/s41598-024-52235-9)
Supplement: Supplementary file 1 — Supplementary Information. [file 41598_2024_52235_MOESM1_ESM.pdf]

New marker for chronic kidney disease progression and mortality in medical-word virtual space

Supplemental file

Eiichiro Kanda[1*], Bogdan I. Epureanu[2], Taiji Adachi[3], Tamaki Sasaki[4], Naoki Kashihara[4]

1. Medical Science, Kawasaki Medical School, Kurashiki, Okayama, Japan.
2. College of Engineering, University of Michigan, Ann Arbor, Michigan, USA.
3. Institute for Life and Medical Sciences, Kyoto University, Sakyo, Kyoto, Japan.
4. Department of Nephrology and Hypertension, Kawasaki Medical School, Kurashiki, Okayama, Japan.

*Corresponding author: Eiichiro Kanda
Email: kms.cds.kanda@gmail.com

Contents

Supplemental Table 1. Vector calculations and CKD-related word vectors. Page 3

Supplemental Table 2. Baseline characteristics of variables included in models. Page 6

Supplemental Table 3. Variables in logistic regression models and effect of each variable on primary outcome. Page 7

Supplemental Table 4. Definition of variables. Page 9

Supplemental Fig. 1. Distributions of selected words. Page 10

Supplemental Fig. 2. Relationships of inner products determined using Models 1 and 2 with outcome risks. Page 11

Supplemental Fig. 3. ROC curve for primary outcome prediction. Page 12

Supplemental Fig. 4. Relationships of inner products determined using Model 3 with outcome risks in subclasses by DM and age. Page 13

Supplemental Fig. 5. Relationships of inner products determined using Model 3 with outcome risks in subclasses by eGFR and proteinuria. Page 14

Supplemental Fig. 6. Virtual space of CKD constructed on the basis of category theory. Page 15

Supplemental Fig. 7. Analysis steps of this study and population for model validation. Page 16

Supplemental Fig. 8. Calculations of a patient vector $\boldsymbol{w}_{pi}$ and an inner product. Page 17

Supplemental Fig. 9. Inner product between vectors. Page 18

Calculation	Word rank	Cos θ
ckd + elderly	*esrd*	0.6672
	older	0.6666
	frail	0.6347
	multimorbidity	0.6040
	geriatric	0.6026
	polypharmacy	0.5972
	cri	0.5961
	eskd	0.5707
	sarcopenia	0.5666
	cvd	0.5626
ckd + proteinuria	*albuminuria*	0.8181
	microalbuminuria	0.7413
	dkd	0.6097
	cri	0.5910
	niddm	0.5865
	macroalbuminuria	0.5687
	lvh	0.5684
	proteinuric	0.5656
	esrd	0.5579
	dm	0.5558
ckd + hypertension	*htn*	0.6724
	hypertensive	0.6645
	cvd	0.6082
	niddm	0.5960
	microalbuminuria	0.5900
	dyslipidemia	0.5861
	lvh	0.5853
	chf	0.5847
	esrd	0.5819
	cri	0.5809

ckd + anemia	*anaemia*	0.8310
	cri	0.6237
	hyperphosphatemia	0.6092
	uraemia	0.6033
	esrd	0.5917
	anemic	0.5856
	uremia	0.5853
	dyslipidemia	0.5801
	lvh	0.5772
	crf	0.5758
ckd + cvd	*esrd*	0.6995
	cardiovascular	0.6544
	lvh	0.6378
	chd	0.6325
	ascvd	0.6323
	niddm	0.6096
	eskd	0.6034
	dm	0.6016
	atherosclerosis	0.5991
	microalbuminuria	0.5990
ckd + malnutrition	*pew*	0.7763
	sarcopenia	0.6897
	cvd	0.6274
	hypovitaminosis	0.6203
	malnourished	0.6177
	uraemia	0.6169
	frailty	0.6122
	pem	0.6077
	uremia	0.5918
	nutritional	0.5834

ckd + *stage*	*esrd*	0.6715
	eskd	0.5732
	cri	0.5650
	predialysis	0.5369
	esrf	0.5349
	cvd	0.5278
	dkd	0.5183
	lvh	0.5107
	crf	0.5090
ckd + *ckd*	*esrd*	0.6329
	cri	0.6241
	cvd	0.6089
	crf	0.6043
	dkd	0.5839
	eskd	0.5715
	lvh	0.5693
	chf	0.5618
	predialysis	0.5617
	hf	0.5566

Supplemental Table 1. Vector calculations and CKD-related word vectors.

To evaluate whether vector calculation can retain the medical meaning, vector calculations and CKD-related words were evaluated using Cosθ of the vectors. $\boldsymbol{w}_{\mathrm{ckd}}$ + $\boldsymbol{w}_{\mathrm{stage}}$ was found to be associated with $\boldsymbol{w}_{\mathrm{esrd}}$ and $\boldsymbol{w}_{\mathrm{eskd}}$, which suggests that $\boldsymbol{w}_{\mathrm{ckd}}$ + $\boldsymbol{w}_{\mathrm{stage}}$ might represent CKD progression. To evaluate scalar multiplication, $\boldsymbol{w}_{\mathrm{ckd}}$ + $\boldsymbol{w}_{\mathrm{ckd}}$ was calculated, and the results were the same as those calculated for the vectors related to $\boldsymbol{w}_{\mathrm{ckd}}$. This indicates that the scalar multiplication of a vector did not change the Cosθs with other vectors.

Abbreviations: htn, hypertension; chd, chronic heart disease; ascvd, atherosclerotic cardiovascular disease; pew, protein-energy wasting; pem, protein-energy malnutrition.

Model 1	
Elderly (%)	12825 (48.5)
Male (%)	13535 (51.2)
CKD stage	
G1 and G2 (%)	19883 (75.2)
G3 (%)	5646 (21.4)
G4 (%)	528 (2.0)
G5 (%)	378 (1.4)
UPCR (g/gCre)	0.17 [0.11, 0.29]
Model 2 (Model 1 +)	
CVD (%)	171 (0.6)
DM (%)	5537 (20.9)
Hypertension (%)	5442 (20.6)
Dyslipidemia (%)	4311 (16.3)
Hypoalbuminemia (%)	1676 (6.3)
Inflammation (%)	1459 (5.5)
Model 3 (Model 2+)	
Anemia (%)	1447 (5.5)
Hyperkalemia (%)	1099 (4.2)
Hyperphosphatemia (%)	241 (0.9)
Hyperuricemia (%)	95 (0.4)
RASI (%)	4578 (17.3)

Supplemental Table 2. Baseline characteristics of variables included in models.
Continuous variables are shown as median (interquartile range). Categorical variables are shown as n (%).
Abbreviations: CKD, chronic kidney disease; UPCR, urinary protein-to-creatinine ratio; CVD, cardiovascular disease; DM, diabetes mellitus; RASI, renin-angiotensin-aldosterone system inhibitors.

Variables	LSM for Model 1	LSM for Model 2	LSM for Model 3
Elderly yes (ref. no)	2.02 (1.49, 2.74) *p*<0.0001	1.54 (1.11, 2.12) *p*=0.0009	1.21 (0.849, 1.73) *p*=0.29
Male yes (ref. no)	1.31 (0.968, 1.77) *p*=0.08	1.24 (0.901, 1.69) *p*=0.19	1.26 (0.898, 1.75) *p*=0.18
CKD yes (ref. no)	166 (123, 222) *p*<0.0001	64.4 (45, 92.1) *p*<0.0001	21.4 (14.1, 32.4) *p*<0.0001
CKD stage (1 stage increase)	0.91 (0.65, 1.26) *p*=0.57	0.91 (0.645, 1.28) *p*=0.59	0.80 (0.56, 1.14) *p*=0.21
UPCR (1 g/gCre increase)	1 (0.999, 1) *p*=0.92	1 (0.999, 1) *p*=0.93	1 (0.999, 1) *p*=0.97
CVD yes (ref. no)		3.12 (1.86, 5.25) *p*<0.0001	2.77 (1.61, 4.79) *p*<0.0001
DM yes (ref. no)		1.87 (1.36, 2.56) *p*<0.0001	1.72 (1.23, 2.41) *p*<0.0001
Hypertension yes (ref. no)		3.36 (2.35, 4.8) *p*<0.0001	2.81 (1.66, 4.74) *p*<0.0001
Dyslipidemia yes (ref. no)		0.351 (0.159, 0.777)	0.44 (0.17, 1.09) *p*=0.076
Hypoalbuminemia yes (ref. no)		2.1 (1.46, 3.02) *p*=0.0009	1.4 (0.959, 2.05) *p*=0.081
Inflammation yes (ref. no)		1.53 (0.903, 2.58) *p*=0.11	1.51 (0.883, 2.57) *p*=0.13
Anemia yes (ref. no)			11.4 (7.87, 16.6) *p*<0.0001
Hyperkalemia yes (ref. no)			2.1 (1.38, 3.21) *p*<0.0001
Hyperphosphatemia yes (ref. no)			3.43 (1.05, 11.2) *p*=0.041
Hyperuricemia yes (ref. no)			0.51 (0.27, 0.95) *p*=0.034
RASI yes (ref. no)			1.01 (0.638, 1.61) *p*=0.96

Supplemental Table 3. Variables in logistic regression models and effect of each variable on primary outcome.

The variables were the same as the inner product models. The values are shown as odds ratio (95% CI) and *p* value. The C-statistics for the prediction of the primary outcome using LSM for Model 1 was 0.887 (95% CI 0.861, 0.912); LSM for Model 2, 0.93 (95% CI 0.913, 0.946); LSM for Model 3, 0.946 (95% CI 0.93, 0.962).

Abbreviations: LSM, logistic regression model; CKD, chronic kidney disease; UPCR, urinary protein-to-creatinine ratio; CVD, cardiovascular disease; DM, diabetes mellitus; RASI, renin-angiotensin-aldosterone system inhibitor; ref, reference.

Variable	Definition
Elderly	Binomial variable. Age of more than 65 years
Male	Binomial variable
CKD stage	Categorical variable. CKD Stages G1 + G2, G3, G4, and G5
Proteinuria	Continuous variable. UPCR level
CVD	Binomial variable. A history of ischemic heart disease, heart failure, severe valvular disease, peripheral arterial disease, cardiovascular surgery, or stroke
DM	Binomial variable. A history of DM and use of DM medicines
Hypertension	Binomial variable. A history of hypertension and use of antihypertensive medicines
Dyslipidemia	Binomial variable. A history of dyslipidemia and use of lipid-lowering medicines
Hypoalbuminemia	Binomial variable. A serum albumin level of less than 3.5 mg/dL
Inflammation	Binomial variable. A white blood cell count of more than $10\times10^3/\mu L$
Anemia	Binomial variable. A hemoglobin level of less than 11 g/dL or use of an erythropoiesis-stimulating agent
Hyperkalemia	Binomial variable. A serum potassium level of more than 5.5 mEq/L or use of medications for hyperkalemia
Hyperphosphatemia	Binomial variable. A serum phosphorus level of more than 6.0 mg/dL or use of phosphate adsorbents
Hyperuricemia	Binomial variable. A serum uric acid level of more than 8.0 mg/dL or use of antihyperuricemics
RASI	Binomial variable. Use of RASI

Supplemental Table 4. Definition of variables.

Abbreviations: CKD, chronic kidney disease; UPCR, urinary protein-to-creatinine ratio; CVD, cardiovascular disease; DM, diabetes mellitus; ESRD, end-stage renal disease; RASI, renin-angiotensin-aldosterone system inhibitors.

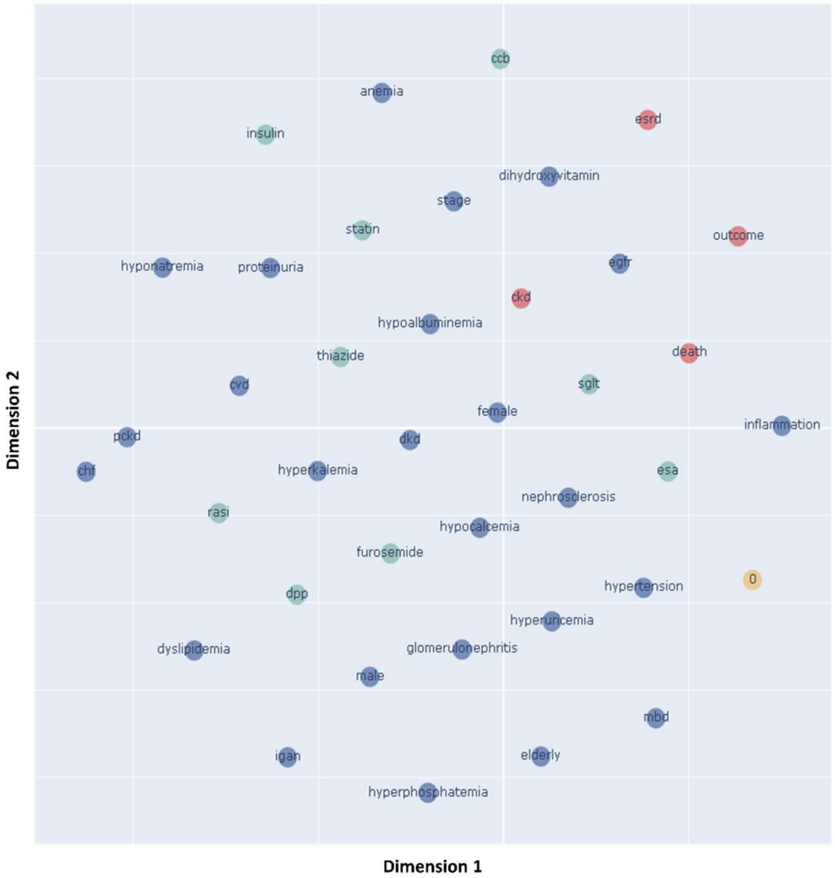

a. Distribution of selected words.

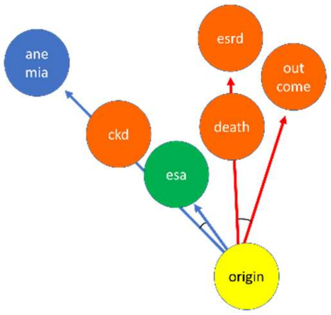

b. Relationship between word vectors.

Supplemental Fig. 1. Distributions of selected words.

a. Distributions of words are shown with their names. Orange, CKD and outcomes; blue, conditions; green, medicines; yellow, the origin.

b. Explanation of Fig. a. The relationship between vectors is evaluated on the basis of the direction (angle θ) and distance from the origin *O*, i.e., Cos θ.

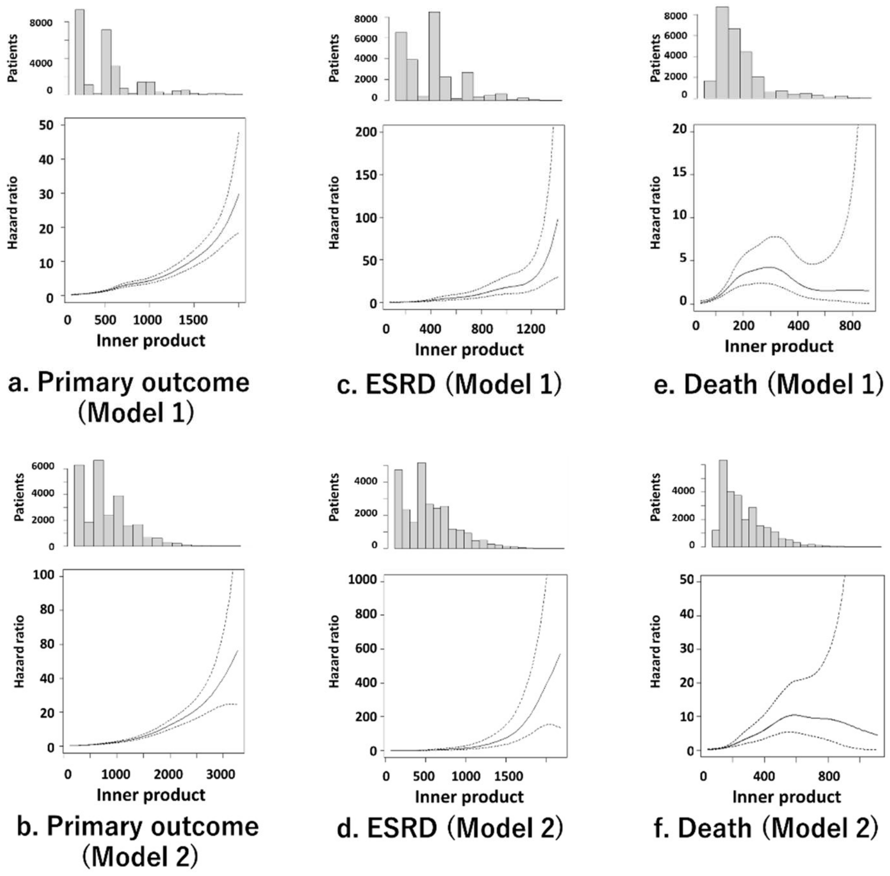

Supplemental Fig. 2. Relationships of inner products determined using Models 1 and 2 with outcome risks.

Histograms of the inner products and the hazard ratios of the outcomes are shown in the upper and lower panels, respectively. Models 1 and 2 show the relationships between the inner product and the primary outcome, between the inner product and ESRD, and between the inner product and death (a to f, $p<0.0001$).

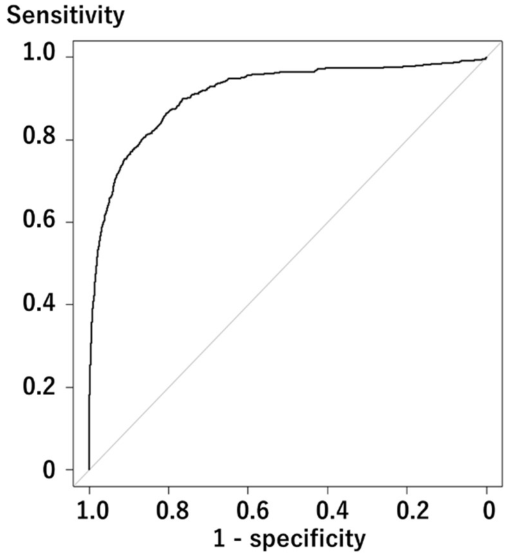

Supplemental Fig. 3. ROC curve for primary outcome prediction.

The accuracy of the prediction of the inner products determined on the basis of Model 3 was evaluated using the ROC curve.

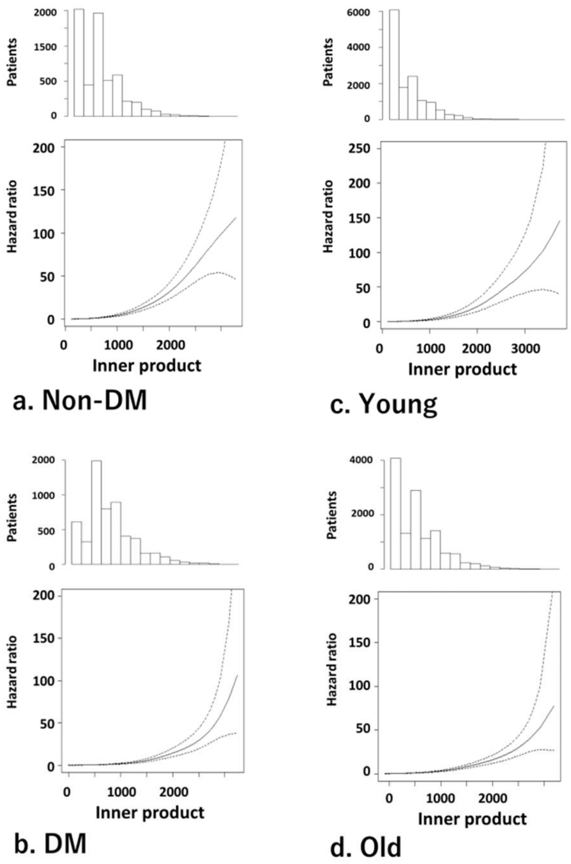

Supplemental Fig. 4. Relationships of inner products determined using Model 3 with outcome risks in subclasses by DM and age.

Histograms of the inner products and the hazard ratios of the primary outcomes are shown in the upper and lower panels, respectively.

Model 3 shows the relationships between the inner product and the primary outcome in subgroups (a to d, $p<0.0001$).

Fig. a. Non-DM.

Fig. b. DM.

Fig. c. Young, younger than 65.

Fig. d. Old, 65 or older.

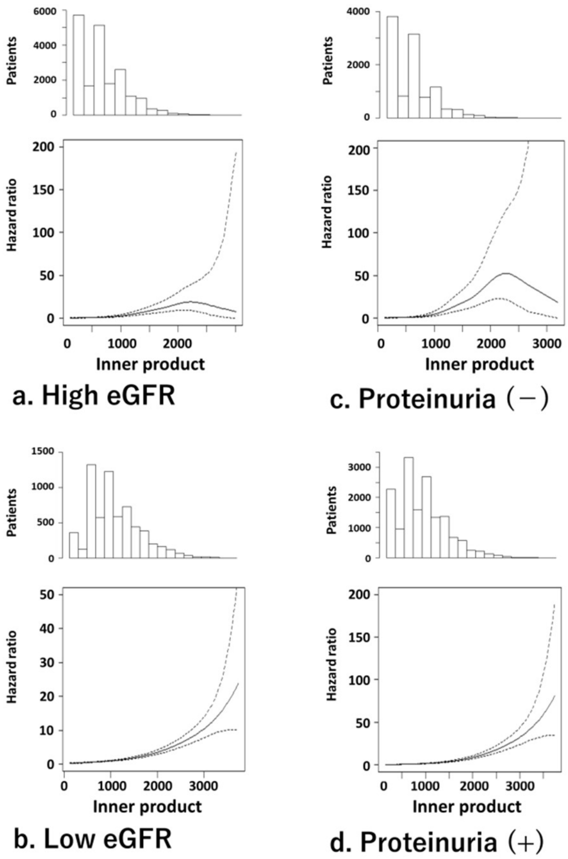

Supplemental Fig. 5. Relationships of inner products determined using Model 3 with outcome risks in subclasses by eGFR and proteinuria.

Histograms of the inner products and the hazard ratios of the primary outcomes are shown in the upper and lower panels, respectively.

Model 3 shows the relationships between the inner product and the primary outcome in subgroups (a to d, $p<0.0001$).

Fig. a. eGFR, 60 mL/min/1.73 m^2 or higher.

Fig. b. eGFR, less than 60 mL/min/1.73 m^2.

Fig. c. Proteinuria, less than 0.15 g/gCr.

Fig. d. Proteinuria, 0.15 g/gCr or higher.

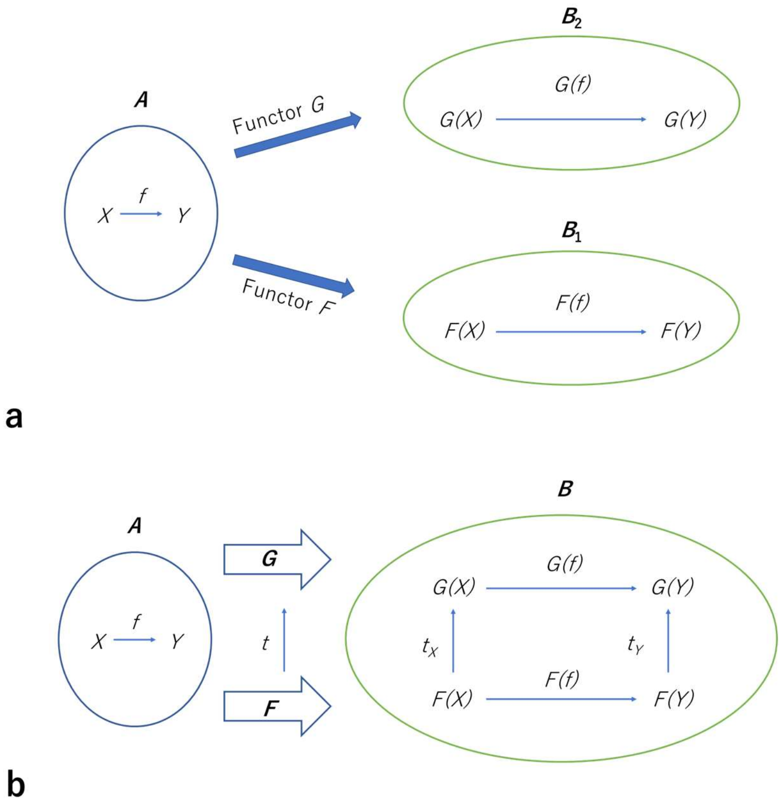

Supplemental Fig. 6. Virtual space of CKD constructed on the basis of category theory.

a. Categories of concept of CKD.

A. Category of concept of CKD.

B_1. Category of data of CKD patients.

B_2. Category of medical papers about CKD.

X. Medical concepts related to pathophysiology of CKD.

Y. Medical concepts related to life and renal prognoses of CKD.

f. Morphism: $X \rightarrow Y$.

F. Functor from A to B_1.

G. Functor from A to B_2.

Functors *F* and *G* are morphisms of categories.

b. Natural transformation of categories of concepts of CKD.

t. Natural transformation from *F* to *G*, $t: F \rightarrow G$.

Categories B_1 and B_2 are treated in category *B*. The natural transformation *t* connects functors *F* and *G*. t_X: $F(X) \rightarrow G(X)$ in *B*. *B* is a virtual space of CKD.

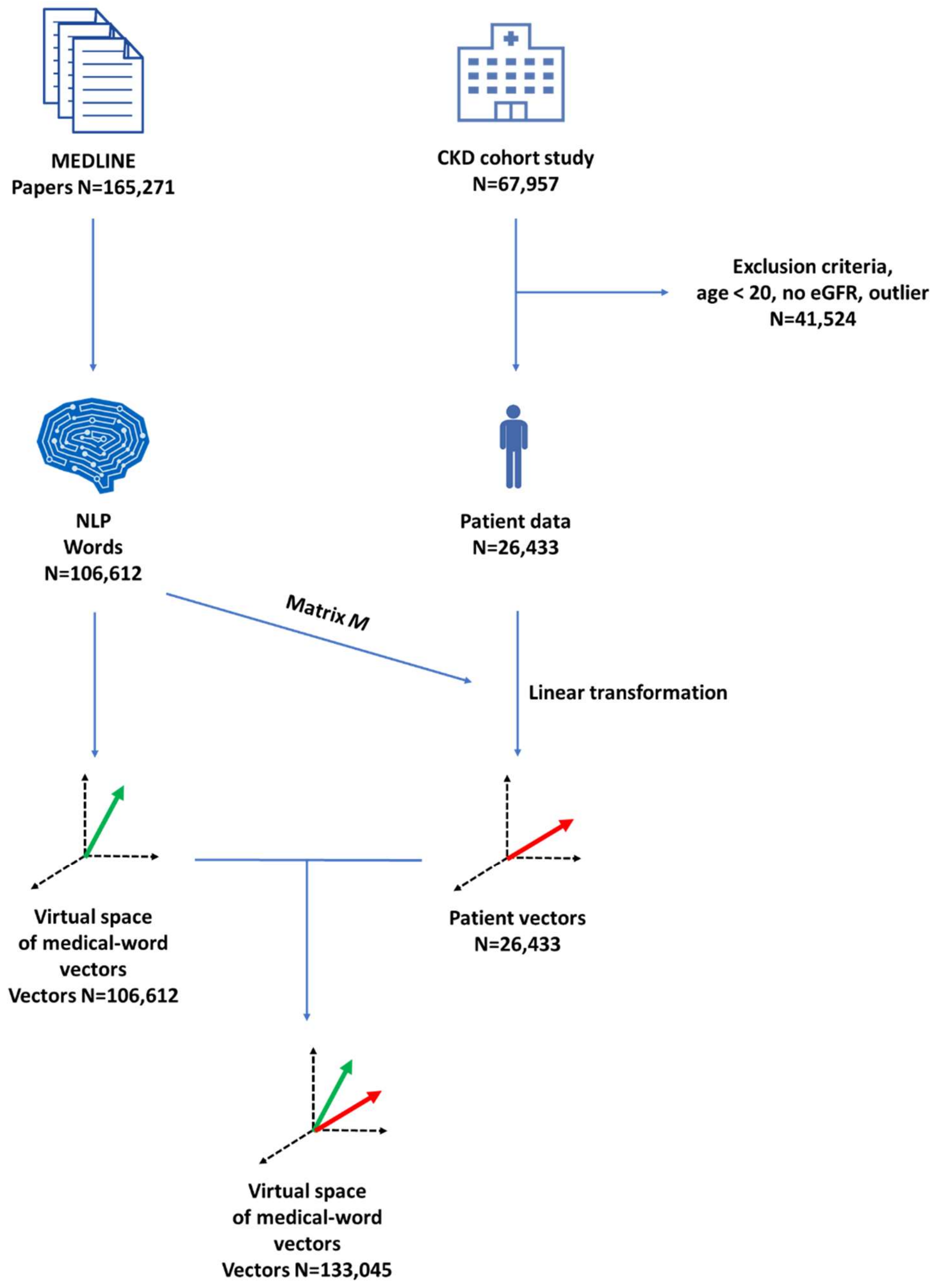

Supplemental Fig. 7. Analysis steps of this study and population for model validation. Texts from MEDLINE were analyzed by NLP, and a virtual space of medical-word vectors was constructed. At the same time, the matrix M was also formed for the linear transformation of patient data. Data from the CKD cohort study were used for the analyses. Then, the patient vectors were mapped into the virtual space.

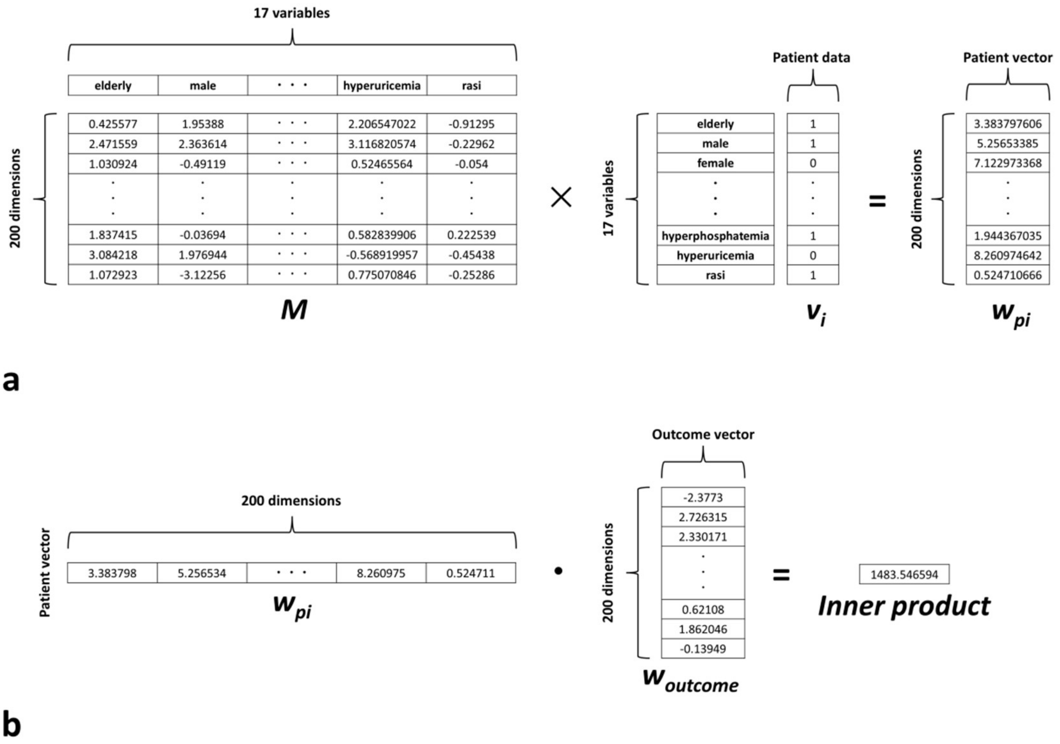

Supplemental Fig. 8. Calculations of patient vector w_{pi} and inner product.

a. Calculation of w_{pi}. M is a matrix composed of medical-word vectors. M transforms the patient data v_i into w_{pi}: $w_{pi} = Mv_i$.

b. Calculation of inner product between w_{pi} and w_{outcome}.

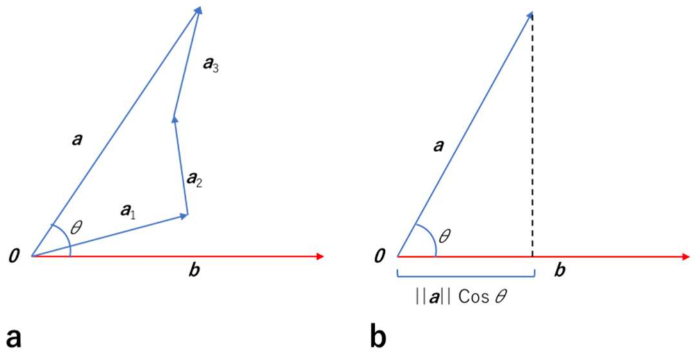

Supplemental Fig. 9. Inner product between vectors.

a. θ is the angle between $\boldsymbol{a}$ and $\boldsymbol{b}$. $\boldsymbol{a}_i$ is a vector of a risk factor, and $\boldsymbol{b}$ is the vector of the outcome. $\boldsymbol{a} = \sum \boldsymbol{a}_i$. The addition of $\boldsymbol{a}_i$ does not provide $\boldsymbol{a}$ closer to $\boldsymbol{b}$ or a smaller θ.

b. Inner product between $\boldsymbol{a}$ and $\boldsymbol{b}$. $inner\ product = ||\boldsymbol{a}||||\boldsymbol{b}||\mathrm{Cos}\vartheta = ||\boldsymbol{a}||\mathrm{Cos}\vartheta \cdot ||\boldsymbol{b}||$. An inner product can be considered as a product of the norm of $\boldsymbol{a}$ mapped to $\boldsymbol{b}$ and that of $\boldsymbol{b}$.
